# Anti-mycobacterial activity of heat and pH stable high molecular weight protein(s) secreted by a bacterial laboratory contaminant

**DOI:** 10.1186/s12934-022-01743-2

**Published:** 2022-01-29

**Authors:** Md. Sajid Hussain, Atul Vashist, Mahadevan Kumar, Neetu Kumra Taneja, Uma Shankar Gautam, Seema Dwivedi, Jaya Sivaswami Tyagi, Rajesh Kumar Gupta

**Affiliations:** 1 School of Biotechnology, Gautam Buddha University, Greater Noida, Uttar Pradesh 201306 India; 2 School of Vocational Studies and Applied Sciences, Gautam Buddha University, Greater Noida, Uttar Pradesh 201306 India; 3 Department of Biotechnology, All India Institute of Medical Sciences, New Delhi, 110029 India; 4 Present Address: Department of Infection & Immunology, Translational Health Science and Technology Institute (THSTI), Faridabad, Haryana 121001 India; 5 Present Address: Department of Microbiology, Bharati Vidyapeeth University, Medical College, Pune, 411043 India; 6 Present Address: Department of Basic and Applied Sciences, NIFTEM, Sonipat, Haryana, 131028 India; 7 Present Address: School of Medicine, Duke University, Durham, NC 27710 USA

## Abstract

**Background:**

Tuberculosis currently stands as the second leading cause of deaths worldwide due to single  infectious agent after Severe Acute Respiratory Syndrome Coronavirus 2 (SARS-CoV-2). The current challenges of drug resistance in tuberculosis highlight an urgent need to develop newer anti-mycobacterial compounds. In the present study, we report the serendipitous discovery of a bacterial laboratory contaminant (LC-1) exhibiting a zone of growth inhibition on an agar plate seeded with *Mycobacterium tuberculosis*.

**Results:**

We utilized microbiological, biochemical and biophysical approaches to characterize LC-1 and anti-mycobacterial compound(s) in its secretome. Based on 16S rRNA sequencing and BIOLOG analysis, LC-1 was identified as *Staphylococcus hominis*, a human bacterial commensal. Anti-mycobacterial activity was initially found in 30 kDa retentate that was obtained by ultrafiltration of culture filtrate (CF). SDS-PAGE analysis of peak fractions obtained by size exclusion chromatography of 30 kDa retentate confirmed the presence of high molecular weight (≥ 30 kDa) proteins. Peak fraction-1 (F-1) exhibited inhibitory activity against *M. bovis* BCG, but not against *M. smegmatis*, *E. coli* and *S. aureus*. The active fraction F-1 was inactivated by treatment with Proteinase K and α-chymotrypsin. However, it retained its anti-mycobacterial activity over a wide range of heat and pH treatment. The anti-mycobacterial activity of F-1 was found to be maintained even after a long storage (~12 months) at − 20 °C. Mass spectrometry analysis revealed that the identified peptide masses do not match with any previously known bacteriocins.

**Conclusions:**

The present study highlights the anti-mycobacterial activity of high molecular weight protein(s) present in culture filtrate of LC-1, which may be tested further to target *M. tuberculosis.* The heat and pH stability of these proteins add to their characteristics as therapeutic proteins and may contribute to their long shelf life. LC-1 being a human commensal can be tested in future for its potential as a probiotic to treat tuberculosis.

**Supplementary Information:**

The online version contains supplementary material available at 10.1186/s12934-022-01743-2.

## Background

The extent and diversity of diseases caused by pathogenic mycobacterial species is of global concern, impacting significantly both human and animal health. Tuberculosis caused by *Mycobacterium tuberculosis* (Mtb), as well as other members of the Mtb complex, remains undeniably a menace to public health [1–4]. Recent statistics from the World Health Organization (WHO) reveal that TB is one of the top 13 causes of death and is anticipated to be second leading cause of death from a single infectious agent after Severe Acute Respiratory Syndrome Coronavirus 2 (SARS-CoV-2) [5]. Globally, an estimated 9.9 million cases of TB (56% men, 33% women and 11% children) were reported in 2020, among which 1.3 million deaths occurred in HIV-negative individuals and an additional 0.214 million deaths in HIV-positive individuals. The emergence of drug-resistant strains has further increased the health security threat due to TB. Globally in 2020, 71% people confirmed with pulmonary TB were resistant to rifampicin, a front line TB drug. Among these, a combined total of 157, 903 cases of MDR/RR-TB/XDR-TB were detected [5]. Thus, it is imperative to devise newer therapeutic tools to address increasing drug resistance and eliminate tuberculosis.

TB drug discovery is hampered by formidable technical challenges owing to the slow growth rate and requirement of Biosafety Level-3 (BSL-3) facilities for bio-safe handling of the TB pathogen. To overcome these challenges, many anti-tubercular drug screening strategies utilize *M. bovis* (BCG) or *M. smegmatis* as a surrogate organism [6, 7]*.* Recently, FDA has approved Bedaquiline (TMC207) as anti-tubercular drug which was first identified during high throughput screening assays using *M. smegmatis* [8]. However, a comparative genome analysis revealed that unlike *M. smegmatis* which lacks ~ 30% of conserved orthologues of Mtb proteins, *M. bovis* differs only by ~ 3% compared to Mtb proteins [7]. Therefore, *M. bovis* (BCG) represents a more suitable model for screening anti-tubercular agents.

Microorganisms are a rich source of antimicrobial compounds [9–11] that are produced as an outcome of various competitive mechanisms including microbial antagonism [12, 13]. Microorganisms may cause inhibition by changing pH, osmotic pressure and surface tension or by producing toxic components, antibiotics, bacteriocins etc. [14–16]. Bacteriocins are ribosomally synthesized antimicrobial peptides or proteins that are produced by various bacterial strains as a strategy to overcome antagonism by invading bacteria that compete for a common environmental niche to survive [17]. Although bacteriocins were previously believed to inhibit the growth of only closely related strains or species, recent studies have shown that they also inhibit the growth of unrelated bacteria [18, 19]. In recent years, bacteriocins have received much attention as a means to combat harmful microbes, especially those resistant to conventional drugs. Some of the promising bacteriocins that are reported to have anti-tubercular activity include Nisin, Lacticin 3147, E50-52, Lassomycin, Lariatins A and B, Nocardithiocin and Sansanmycin [20–24]. *Staphylococcus* species, which are the dominant bacterial colonisers of skin have been found to produce bacteriocins and antimicrobial peptides [25, 26], many of which inhibit *S. aureus* infections [27–30]. Besides having anti-*S. aureus* activity, bacteriocins from Staphylococci have been reported to inhibit other pathogens including Mtb [31–33].

A serendipitous finding in our laboratory led to the detection of a bacterial contaminant which caused a prominent zone of growth inhibition of Mtb on a 7H11 solid agar plate. In the present study, the contaminant bacterial strain was identified as *Staphylococcus hominis*. Various microbiological, biochemical and biophysical approaches were utilized to characterize the anti-mycobacterial principle(s) secreted by this bacterial strain.

## Results

### Antagonistic spectrum of LC-1

**Fig. 1:**
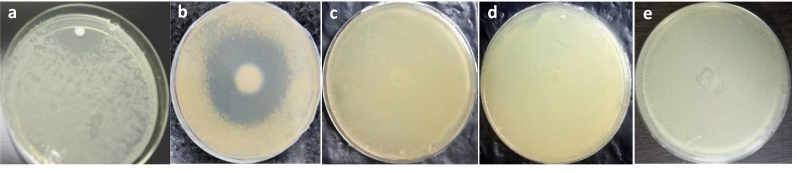
Antagonistic spectrum of LC-1 against various bacterial strains by spot bioassay. **a** *M. tuberculosis* H37Rv; **b** *M. bovis* BCG; **c** *M. smegmatis*; **d** *E. coli*; **e** *S. aureus*

A significant zone of inhibition was serendipitously observed around a laboratory contaminant (LC-1) on a 7H11 agar plate seeded with Mtb H37Rv culture (Fig. 1a), suggesting that LC-1 secretes a potential anti-mycobacterial component. LC-1 was further screened for its growth inhibitory property against *M. smegmatis, M. bovis* BCG, *E. coli* and *S. aureus*. The antagonistic spectrum of LC-1 was assessed by spot bioassay (Fig. 1). LC-1 exhibited a promising antagonistic property against *M. bovis* BCG, quite similar to Mtb H37Rv strain, but not against *M. smegmatis*, *E. coli* and *S. aureus*. Since Mtb is a pathogenic bacterium and requires BSL-3 facility for its handling, we utilized *M. bovis* BCG as a surrogate strain to characterize the active principle in a BSL-2 laboratory set up.

### Identification and characterization of LC-1

LC-1 produced medium-sized, smooth, opaque white colonies on an LB agar plate. It was characterized to be Gram positive, non-motile, non-spore forming cocci present in clusters, positive for nitrate reduction and catalase test. It was found to hydrolyse Tween 20 but not Tween 40 and Tween 80. The strain was able to produce acid from glucose, lactose, sucrose and glycerol. LC-1 showed growth between temperatures ranging from 15 to 42 °C, pH ranging from pH 5.0 to 10.5 and salt concentration ranging from 2 to 10%. The various morphological, biochemical and physiological properties of LC-1 are presented in Tables 1 and 2. Based on interpretation of the BIOLOG database, LC-1 was identified as *Staphylococcus hominis.* Genomic analysis of 16S rRNA gene sequence of LC-1 further confirmed the strain as *Staphylococcus hominis*.

**Table 1:** Morphological and physiological properties of LC-1

Test	Result	Test	Result
**a. Colony morphology**			
Configuration	Round	37 °C	+
Margin	Entire	42 °C	+
Elevation	Raised	55 °C	−
Surface	Smooth		
Pigment	White	**c. Growth at pH**	
Opacity	Opaque	5	+
Gram's reaction	+	6	+
Cell shape	Cocci	7	+
Size (µm)	1.2–1.4 µm	8	+
Spore(s)	−	9	+
Motility	−	10.5	+
**b. Growth at temperature**		**d. Growth in NaCl (%)**	
4 °C	−	2	+
15 °C	+	4	+
20 °C	+	6	+
30 °C	+	10	+

**Table 2:** Biochemical properties of LC-1

Test	Results	Test	Results
Growth on MacConkey agar	−	Tween 20 hydrolysis	+
Indole test	−	Tween 40 hydrolysis	−
Methyl red test	−	Tween 80 hydrolysis	−
Voges Kauer test	−	Arginine dihydrolase	−
Citrate utilization	−		
H_2_S production	−	**Acid production from**	
Gas production	−	Glucose	+
Casein hydrolysis	−	Lactose	+
Esculin hydrolysis	−	Sucrose	+
Gelatin hydrolysis	−	Arabinose	−
Starch hydrolysis	−	Salicin	−
Urea hydrolysis	−	Mannose	−
Nitrate reduction	+	Mannitol	−
Catalase test	+	Xylose	−
Oxidase test	−	Glycerol	+

### Production and purification of anti-mycobacterial component from LC-1 culture filtrate

**Fig. 2:**
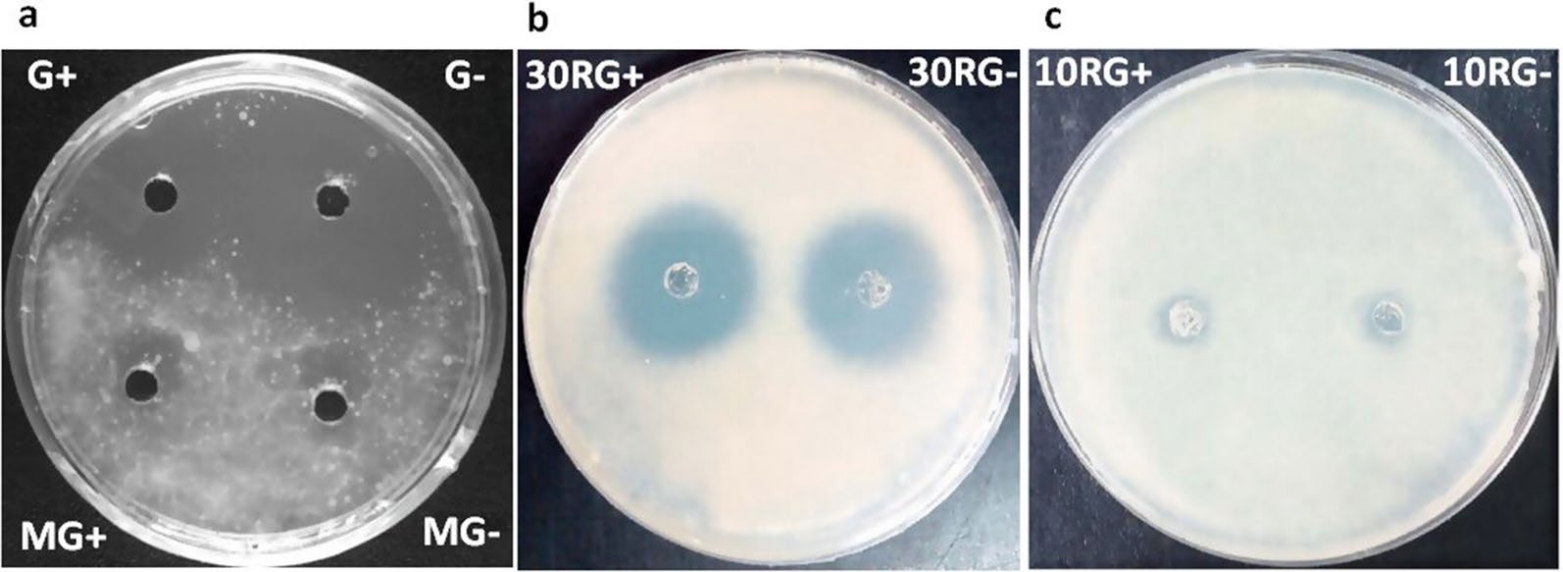
Anti-mycobacterial activity of culture filtrate (CF) and its molecular weight cut-off fractions against *M. bovis* BCG. **a** CF from culture grown in Glycerol enriched medium (G+) and without glycerol (G−), MG+ & MG− represent respective media controls; **b** 30 kDa retentates of G+ (30RG+) and G− (30RG−); **c** 10 kDa retentates of 30FG+ (10RG+) and 30FG− (10RG−). 30FG+ and 30FG− are 30 kDa filtrates of G+ and G− respectively

The Culture Filtrate (CF) of LC-1 that was grown in nutrient broth was first screened for anti-mycobacterial activity against *M. bovis* BCG. CF showed promising inhibitory activity, whereas no activity was reported in media control, MC (Fig. 2a), establishing that LC-1 secretes some anti-mycobacterial substance/s.

**Fig. 3:**
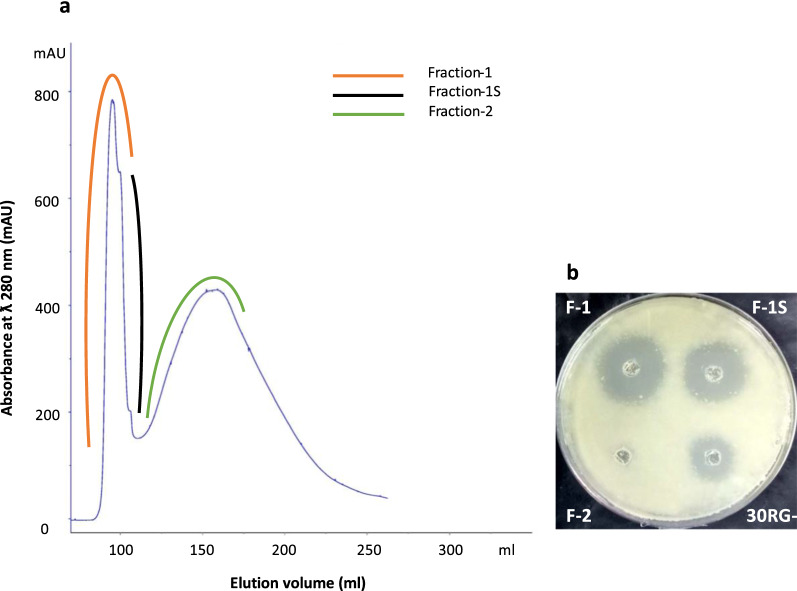
Purification and characterization of 30RG−. **a** Gel filtration profile of 30RG− using Sephacryl S-100 column; **b** anti-mycobacterial activity of 30RG−, F-1 (Fraction-1), F-1S (F-1 Shoulder fraction) and F-2 (Fraction-2)

The CF of LC-1 grown in nutrient broth supplemented with Glycerol (G+) and without glycerol (G−) were found to be acidic (pH 4.5–5.0) and basic (pH 7.2–7.5), respectively. However, both G+ and G− exhibited comparable inhibitory activity against *M. bovis* BCG (Fig. 2a), indicating the anti-mycobacterial activity to be pH independent. G+ and G− were further subjected to sequential ultrafiltration using centrifugal filter device with cut-off of 30 kDa followed by 10 kDa to separate the anti-mycobacterial proteins based on their molecular weight. Unlike 10 kDa retentates (10RG+ and 10RG−), a significant anti-mycobacterial activity was noted in 30 kDa retentates (30RG+ and 30RG−), suggesting the anti-mycobacterial component to be of high molecular weight (Fig. 2b, c). Since no significant difference was observed between anti-mycobacterial activity of 30RG+ and 30RG− retentates (Fig. 2b), we proceeded with 30RG− for further purification of active principle by gel permeation chromatography. The peaks so obtained, namely Fraction-1 (F-1), F-1 shoulder fraction (F-1S) and Fraction-2 (F-2), were tested for activity against *M. bovis* BCG (Fig. 3a). F-1 exhibited predominant activity among all the tested fractions (Fig. 3b**)**, so it was taken forward for further characterization.

### Effect of protease, heat and pH treatments on anti-mycobacterial activity of F-1

**Fig. 4:**
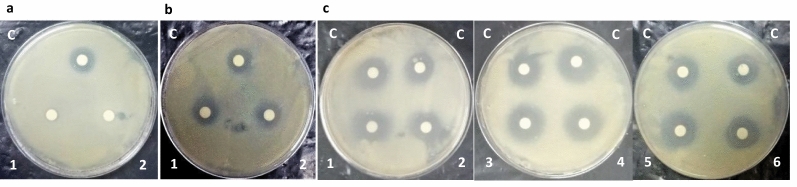
Effect of different physical and chemical treatments on anti-mycobacterial property of F-1. **a** Proteolytic enzymes treatment—C: untreated F-1 (control); 1: treated with α-chymotrypsin; 2: treated with Proteinase K; **b** heat treatment—C: untreated F-1 (control); 1: treated for 30 min; 2: treated for 60 min; **c** pH treatment—1: treated with buffer of pH 3.0; 2: treated with buffer of pH 4.5; 3: treated with buffer of pH 6.0; 4: treated with buffer of pH 7.5; 5: treated with buffer of pH 9.0; 6: treated with buffer of pH 10.5; C: untreated F-1 (control)

F-1 was found to be highly sensitive to Proteinase-K and α-chymotrypsin enzymes treatment as its anti-mycobacterial activity was completely lost post-treatment, confirming its proteinaceous nature (Fig. 4a). Heat stability test revealed that upon heat treatment at 100 °C for 60 min, anti-mycobacterial protein/s in F-1 retained approximately 90–100% of activity with respect to untreated control (Fig. 4b) suggesting them to be thermally stable. Beside this, F-1 was also found to stably exhibit anti-mycobacterial activity against *M. bovis* BCG even after a long storage (~12 months) at − 20 °C (data not shown). F-1 was also found to be active over a wide range of acidic and alkaline pH (Fig. 4c).

### Antimicrobial spectrum of F-1

**Fig. 5:**
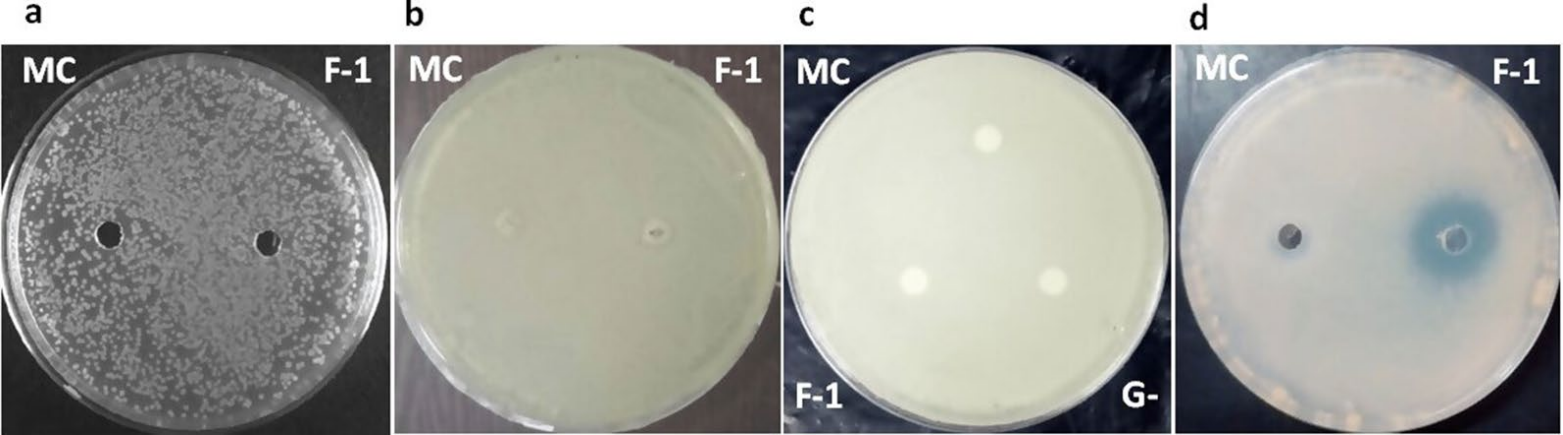
Antimicrobial activity of F-1 against different bacteria. **a** *E. coli*; **b** *S. aureus*; **c** *M. smegmatis*; **d** *M. bovis* BCG. MC: media control; F-1: Fraction-1; G−: culture filtrate without Glycerol

F-1 exhibited promising anti-mycobacterial activity against *M. bovis* BCG strain with a prominent zone of inhibition of ~ 24 mm in diameter (Fig. 5d). However, no activity was observed against *M. smegmatis* (Fig. 5c), *E. coli* DH5α (Fig. 5a) and *S. aureus* (Fig. 5b), indicating that among the strains that were tested, F-1 specifically inhibits the growth of *M. bovis* BCG. Consistent with the result of disc diffusion assay, treatment of *M. bovis* BCG culture with fraction F-1 (300 µg/ml of protein) resulted in a statistically significant (P < 0.05) inhibition of mycobacterial growth in 7H9-ADS liquid media as compared to untreated culture control. However, no significant inhibition was observed in buffer control (Additional file 1: Fig. S1).

### MALDI-TOF MS/MS analysis of F-1 and F-1S fraction

**Fig. 6:**
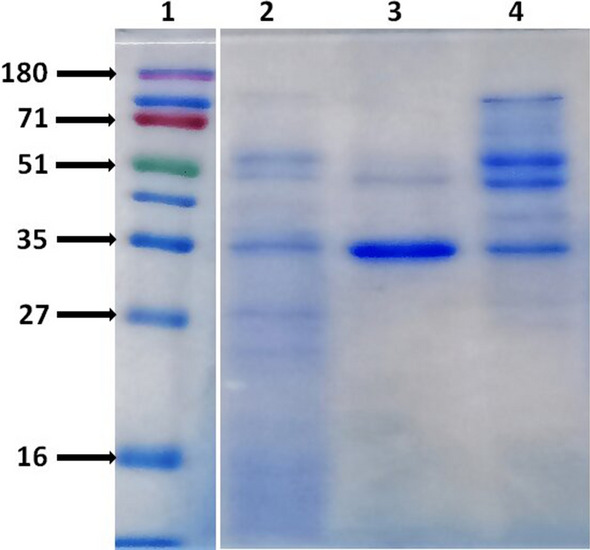
SDS-PAGE analysis of 30RG− and its various cut-off fractions. Lane 1: molecular weight Marker; lane 2: 30RG−; lane 3: Fraction-1 (F-1); lane 4: F-1 shoulder fraction (F-1S)

SDS-PAGE analysis of F-1 and F-1S indicated the presence of proteins with molecular weight above 30 kDa (Fig. 6). Fraction F-1 consists of a prominent band of ~ 35 kDa (Fig. 6, Lane 3), therefore this band was excised from SDS-PAGE gel for MALDI-TOF MS/MS analysis. The database search using MASCOT search engine revealed that the ~ 35 kDa protein band of fraction F-1 exhibited best match with six proteins of molecular weight ranging between 34.934 and 64.548 kDa and MASCOT scores of 56–964 (Table 3). The cell membrane protein SphX (gi|22846002) of molecular weight 34.934 kDa, from *Staphylococcus hominis* SK119, was the closest match with a MASCOT score of 964.

**Table 3:** Details of protein (~ 35 kDa) of F-1 fraction identified through MALDI-TOF MS/MS analysis

S. no.	Protein reference	Protein name	Source organism	Molecular weight (kDa)	Mascot score	Matched sequence	Cellular localization
1	gi|22846002	SphX	*Staphylococcus hominis* SK119	34.934	964	29	Cytoplasmic membrane
2	gi|70726523	Thioredoxin reductase	*Staphylococcus haemolyticus* JCSC1435	35.097	290	9	Cytosol/mitocondria
3	gi|15923133	Alkylphosphonate ABC transporter	*Staphylococcus aureus* subsp. aureus Mu50	35.038	69	3	Cytoplasmic membrane
4	gi|70725319	Hypothetical protein SH0318	*Staphylococcus haemolyticus* JCSC1435	35.104	69	3	Periplasm
5	gi|228469577	Conserved hypothetical protein	*Porphyromonas uenonis* 60–3	64.548	56	3	Unknown
6	gi|189183631	Hypothetical protein OTT_0724	*Orientia tsutsuga*mushi str. Ikeda	44.775	56	3	Unknown

F-1S fraction contained a greater number of visible protein bands as compared to F-1 (Fig. 6, Lane 4) and this fraction also exhibited substantial inhibition against *M. bovis* BCG (Fig. 3b). Therefore, the complete fraction (in solution) was subjected to MALDI-TOF MS/MS analysis which revealed the presence of 100 proteins with MASCOT scores ranging between 38 and 444. The top 10 proteins with high MASCOT scores (240–444) are listed in Table 4. The protein, SphX, was common in both fractions F-1 and F-1S. Since, SphX is a cell membrane protein, its presence in F-1 and F-1S may be attributed to partial lysis of LC-1 during bacterial culturing. Database search also showed the presence of some hypothetical proteins in both F-1 and F-1S. MASCOT search in the NCBInr database revealed that the peptides fragments did not match with any previously known bacteriocins suggesting the presence of some novel bacteriocin/therapeutic protein(s) contributing to anti-mycobacterial activity of F-1 and F-1S. The amino acid sequences of the proteins listed in Tables 3 and 4 are provided in Additional file 1: Fig. S2.

**Table 4:** Detail of top 10 proteins in F-1 shoulder fraction (F-1S) identified through MALDI-TOF MS/MS analysis

S. no.	Protein reference	Protein name	Source organism	Molecular weight (kDa)	Mascot score	Matched sequence	Cellularlocalization
1	gi|22847567	Lipase	*Staphylococcus hominis* SK119	62.029	444	7	Cytoplasmic membrane
2	gi|228474296	Dihydrolipoyl dehydrogenase	*Staphylococcus hominis* SK119	49.716	431	6	Cytosol/mitocondria
3	gi|228474409	Phosphopyruvate hydratase	*Staphylococcus hominis* SK119	47.154	413	9	Cytoplasm
4	gi|228476002	SphX	*Staphylococcus hominis* SK119	34.934	384	7	Cytoplasmic membrane
5	gi|228475766	Alkaline phosphatase 3	*Staphylococcus hominis* SK119	53.395	381	7	Cytoplasmic membrane
6	gi|15924086	Dihydrolipoamide dehydrogenase	*Staphylococcus hominis* SK119	49.421	347	4	Cytosol/mitocondria
7	gi|228474919	Purine nucleoside phosphorylase	*Staphylococcus hominis* SK119	25.905	320	5	Cytosol
8	gi|228476046	DNA binding protein HU 1	*Staphylococcus hominis* SK119	9.650	283	5	Cytoplasmic membrane
9	gi|70725402	Alkaline phosphatase III precursor	S*taphylococcus haemolyticus* JCSC1434	53.385	244	6	Cytoplasmic membrane
10	gi|224475921	2-Phospho-d-glycerate hydrolase (enolase)	*Staphylococcus carnosus* subs. carnosus TM300	47.240	240	3	Cytosol/cytoplasmic membrane

## Discussion

*Staphylococcus* species have been reported previously as a dominant contaminant in Mtb cultures [34]. Their faster growth rate tends to physically mask Mtb growth, and thus may compromise Mtb diagnosis in clinical samples [34]. The normal skin microbiome is dominated by *Firmicutes* (53.3%), the majority (24.2%) of which belong to *Staphylococcus* genus [35]. Interestingly, the disruption of microbial composition has been reported to occur in skin conditions such as leprosy, psoriasis, atopic dermatitis, impetigo, acne and impaired healing of chronic wounds [36–38], which points towards the protective role of normal skin microbiota. *Staphylococcus* species confer protection against pathogenic microorganisms like *M. leprae* by producing bioactive compounds, including antimicrobial peptide/proteins/short chain fatty acids during colonizing on the skin [36, 39, 40].

In the present study, a serendipitous bacterial contaminant, LC-1, was observed on a 7H11 agar plate which exhibited antagonistic activity against Mtb. It was identified as *Staphylococcus hominis* on the basis of 16S rRNA gene sequencing and BIOLOG assay. LC-1 was found to be antagonistic to *M. bovis* BCG and Mtb but not to *M. smegmatis*, *S. aureus* and *E. coli*. This is consistent with the published report of the ability of *S. hominis* to inhibit Mtb H37Rv [41]. Unlike LC-1, *S. hominis* MBBL 2–9 and *Staphylococcus* sp. DBOCP06 were found to exhibit antagonistic property against methicillin resistant *S. aureus* and *E. coli* [30, 42]. This could be attributed to differences at strain level resulting in secretion of different anti-microbial agents.

LC-1 was initially detected on a 7H11 agar plate containing 0.5% glycerol. Biochemical characterization of LC-1 revealed that it was able to ferment glycerol, glucose, sucrose and lactose to produce organic acids. Since organic acids produced by bacterial fermentation of various carbon sources are known to possess antimicrobial activity [43, 44], we investigated the contribution of any acid if produced due to LC-1-mediated fermentation of glycerol to its anti-mycobacterial activity. As expected, G+ prepared from cultures grown in presence of glycerol exhibited acidic pH (4.5–5.0) whereas G− prepared from cultures grown in absence of glycerol, exhibited an alkaline pH (7.2–7.5). However, no significant difference in the anti-mycobacterial activities of G+ and G− was noted (Fig. 2a), which confirmed this activity to be independent of sugar fermentation and pH effects.

The anti-mycobacterial principle present in G− was partially purified using ultrafiltration followed by size permeation chromatography. Only 30 kDa retentate (30RG−) inhibited the growth of *M. bovis* BCG while no inhibition was noticed in 10 kDa retentate (10RG−), suggesting anti-mycobacterial protein/s to have molecular weight > 30 kDa (Fig. 2b, c). Gel permeation chromatography of 30RG− retentate yielded Fractions F-1 and F-1S with significant anti-mycobacterial activity (Fig. 3a, b). SDS-PAGE analysis of F-1 and F-1S confirmed that anti-mycobacterial protein/s in Fractions F-1 and F-1S have molecular weight > 30 kDa (Fig. 6). Previously, majority of the antimicrobial proteins isolated from different strains of *S. hominis* were low molecular weight peptide/proteins [29, 31–33], which indicates that LC-1 may be secreting a new class of antimicrobial proteins.

The partially purified anti-mycobacterial fraction F-1 was heat and pH stable but it was sensitive to Proteinase K and α-chymotrypsin, confirming the proteinaceous nature of active principle (Fig. 4a). The heat and pH stability properties of anti-mycobacterial principle(s) in Fraction F-1 may prove to be useful in imparting long shelf life at room temperature and also constitutes an important feature for any drug that is administered orally, as it remains protected from the acidic and alkaline pH of gastrointestinal tract [45]. Like LC-1, active fraction F-1 also exhibited a narrow antimicrobial spectrum (Fig. 5) which may prove to be advantageous over traditional antibiotics that results in undesirable disruption of host microbiome [46]. Unlike fraction F-1 of culture filtrate from LC-1, which exhibited a zone of inhibition of diameter ~ 24 mm against *M. bovis* BCG (Fig. 5d), the culture filtrate from different strains of *Lactobacillus plantarum* exhibited a zone of inhibition of (ranging between 9 and 22 mm diameter) against *Mycobacterium B5* strain [47]. This difference in activity may be attributed to differences in culture conditions, type of strains, purification status and the concentration of anti-mycobacterial substances present.

Some of the proteins detected in fractions F-1 and F-1S by mass spectrometry (MS) analysis have been previously reported to possess antimicrobial activity. The major protein band (~ 35 kDa) of F-1 exhibited maximum number of peptide match to protein SphX from *S. hominis* SK119, a membrane-bound protein that is involved in phosphate transport across the cytoplasmic membrane [48]. Fraction F-1S was found to contain several proteins, including alkaline phosphatase which is produced by diverse bacterial genera [49, 50]. Various hydrolases including alkaline and acid phosphatases in alveolar lining fluid have been shown to alter Mtb cell envelope during Mtb infection in humans, resulting in the release of cell envelope fragments that modulate macrophages to control Mtb infection in an IL-10 dependent manner [51]. However, it was also noted that alkaline phosphatase by itself does not impair Mtb growth [51]. In contrast, antibacterial activity of alkaline phosphatase from *E. coli* and alkaline phosphatase-like protein from *Naja ashei* venom was reported against *P. aeruginosa* and *S. epidermidis*, respectively [52, 53]. Another protein found in Fraction F-1S matches with peptides corresponding to lipase of *S. hominis* SK119. Lipases have also been reported to possess therapeutic potential against Herpes simplex virus-1 and antibacterial activity against *S. epidermidis* [54].

## Conclusions

Bacteriocins/antimicrobial peptides produced by human microbiota have gained significant attention owing to their novel mechanism of action and narrow spectrum of activity and thus may prove to be valuable biopharmaceutical agent against drug resistant pathogens. The present study identifies a serendipitous laboratory contaminant, LC-1 as *S. hominis*, a human commensal, exhibiting specific antagonistic activity against Mtb and *M. bovis* BCG but not against *E. coli*, *M. smegmatis* and *S. aureus*, suggesting it to be possibly non-toxic to human microbiota. This suggests that LC-1 holds probiotic potential which may be tested further to target Mtb.

The partially purified fractions F-1 and F-1S from LC-1 culture filtrate consist of promising high molecular weight anti-mycobacterial protein/s which may be dissected further to develop a therapeutic agent against Mtb to synergize the existing anti-tubercular drugs.

## Methods

### Bacterial strains and growth conditions

The bacterial strains used in this study are listed in Table 5. All mycobacterial strains were cultured in 7H9 Middlebrook broth containing 0.2% glycerol (G) and 0.1% Tween 80 supplemented with 10% ADS (5% Albumin, 2% Dextrose, 0.85% Saline). Anti-mycobacterial assays were performed on Middlebrook’s 7H11 agar medium supplemented with 10% ADS and 0.5% G (Sigma Aldrich, USA). Luria–Bertani broth and agar was used to culture  *E. coli* and *S. aureus*. Nutrient broth was used to culture LC-1. All the media were purchased from BD bioscience and Hi-media and prepared according to the manufacturer’s recommendation.

**Table 5:** Bacterial strains used in this study

Bacterial strain	Culture medium and temperature	Source
Lab contamination strain (LC-1)	NB at 37 °C	A
*Mycobacterium tuberculosis* H37Rv	7H9 at 37 °C	B
*Mycobacterium bovis* BCG strain	7H9 at 37 °C	C
*Mycobacterium smegmatis* mc^2^ 155	7H9 at 37 °C	D
*Escherichia coli* DH5α	LB at 37 °C	E
*Staphylococcus aureus* (clinical strain)	LB at 37 °C	F

NB: nutrient broth; 7H9: 7H9 broth supplemented with 0.2% glycerol, 0.1% Tween 80 and 10% OADC/ADC; LB: Luria Bertani broth; A: Department of Biotechnology, All India Institute of Medical Sciences, New Delhi, India; B: Kind gift from Dr. Richard F. Silver, Case Western Reserve University, Cleveland, Ohio, USA; C: Kind gift from Dr. Calvin Boon, Mycobacterium Biology Laboratory, Institute of Molecular and Cell biology, Singapore; D: Kind gift from Dr. D. Chatterji, Indian Institute of Science, Bangalore, India; E: Invitrogen Inc., Carlsbad, CA, USA; F: Department of Microbiology, All India Institute of Medical Sciences, New Delhi, India

### Isolation of bacterial strain with anti-tubercular activity

The bacterial strain harbouring anti-tubercular activity originally appeared as a contaminant and produced a clear zone of growth inhibition on a lawn of Mtb cultured on 7H11 agar plate at the Department of Biotechnology, AIIMS, India. The contaminant bacterial strain was aseptically picked and homogenised in 1 ml nutrient broth (Peptone: 5 g/l, Beef extract: 1.5 g/l, Yeast extract: 1.5 g/l Sodium chloride: 5 g/l) and finally streaked on Luria–Bertani agar (Peptone: 10 g/l; Yeast extract: 5 g/l; Sodium chloride: 10 g/l, Agar: 15 g/l). The plate was incubated at 37 °C for 24 h. After incubation, a single colony was picked and cultured aerobically in 10 ml nutrient broth at 37 °C, in shaking incubator for overnight.

### Identification and characterization of LC-1

LC-1 was first characterized based on morphological, biochemical and physiological properties followed by BIOLOG at Microbial Type Culture Collection and Gene Bank (MTCC), Institute of Microbial Technology, Chandigarh, India (on charge basis). Morphological characteristics of LC-1 were determined by colony morphology, Gram reaction, mortality and spore forming ability using microscopic observation (Table 1). Biochemical characteristics of the strain were evaluated using various biochemical tests (Table 2). The strain was also investigated to evaluate fermentation of different carbohydrates. Physiological properties of the strain were evaluated using growth at different temperatures (4 °C–55 °C), pH (5.0–10.5) and NaCl concentration (2–10%). LC-1 isolate was further identified using BIOLOG (Biolog, Inc MicroLog, Hayward, CA, USA; http://www.biolog.com), that uses automated biochemical methodologies to test microorganism's ability to utilize different carbon sources. The test yields characteristic pattern of purple wells which constitute a metabolic fingerprint of the strain being tested. The assay was performed in Biolog GP2 MicroPlate following the procedure provided by the manufacturer. LC-1 was further characterized using 16S rRNA gene sequencing. PCR amplification of 16S rRNA gene was carried out using forward primers (FU1): 5′-CCA GCA GCC GCG GTA ATA CG-3′ and reverse primer (RU2): 5′-ATC GGC TAC CTT GTT ACG ACT TC-3′ using genomic DNA as template. The amplified PCR product (996 bp) was quantified and confirmed on agarose gel (1.5%) and subjected to DNA sequence analysis.

### Antagonistic spectrum of LC-1

Spot bioassay was carried out to screen antagonistic spectrum of LC-1 against *M. smegmatis*, *M. bovis* BCG, *E. coli* and *S. aureus* with slight modification [55]. All the bacterial strains were grown in their appropriate media at 37 °C, sub-cultured in fresh broth and allowed to grow to an OD_595_ of approximately 0.4–0.5. The cultures were diluted in their respective broth to obtain final O.D_595_ of 0.025 for *M. bovis* BCG, 0.0025 for *M. smegmatis*, and 0.000025 for *E. coli* and *S. aureus*. The uniform lawns of test strains were prepared by seeding 750 µl of diluted cultures individually on their respective agar plates. Five microliters of overnight LC-1 culture were spotted at the center of the plate containing lawns culture of *M. smegmatis*, *E. coli* and *S. aureus* and incubated at 37 °C for 24–48 h. Agar plates containing *M. bovis* BCG lawn were initially incubated for 2 days at 37 °C, followed by spotting 5 µl of LC-1 culture on the lawn and incubating the plates at 37 °C for 15–21 days. The inhibitory activity against the plated bacteria was evaluated by visualizing the zone of inhibition around LC-1 colony. LC-1 exhibiting antagonistic property was picked aseptically and inoculated into nutrient broth, grown overnight at 37 °C and stored in 18% glycerol at − 80 °C for further use.

### Investigation of anti-mycobacterial activity of the culture filtrate

LC-1 was initially grown overnight in sterilized nutrient broth at 37 °C under shaking (180 rpm), and 1% inoculum of this culture was added to 1200 ml nutrient broth and incubated for 48 h at 37 °C and 180 rpm. The cells were centrifuged at 5000 rpm for 15 min at 4 °C, and the culture supernatant was filtered using 0.45 µm membrane filter (Millex GV filter, Millipore). The cell-free culture filtrate (CF) was concentrated tenfold (by volume) through lyophilization and stored at − 20 °C for further use. Nutrient broth without culture was processed in parallel and labelled as Media control (MC). CF and MC were examined for anti-mycobacterial activity against *M. bovis* BCG strain using agar-well diffusion assay [55]. Briefly, a secondary culture of *M. bovis* BCG was grown to an O.D_595_ of 0.4–0.5 in 7H9-ADS media and diluted as above to obtain final O.D_595_ of O.025. A uniform lawn was prepared by seeding 750 µl of diluted culture, wells were prepared using a sterilized cork borer and 100 µl of 0.22 µm filter-sterilized CF and MC were added to respective wells. The plates in duplicate were incubated at 37 °C for 15–21 days. Anti-mycobacterial activity of CF was evaluated by observing zone of inhibition around the well and comparing it with that of MC.

### Production and purification of anti-mycobacterial product

The production of anti-mycobacterial component from LC-1 was tested in two different culture conditions, by growing LC-1 in nutrient broth in absence or presence of Glycerol (0.5%) under shaking conditions of 180 rpm at 37 °C for 48 h. The secondary culture was centrifuged at 5000 rpm for 15 min at 4 °C and the pellets were removed. The culture filtrate (CF) was passed through a 0.45 µm membrane filter (Merck Millipore, USA) using filtration assembly (Millipore, USA) to completely remove the bacterial cells. CF obtained from mycobacterial culture grown in medium with and without Glycerol were labelled as G+ and G−, respectively.

**Fig. 7:**
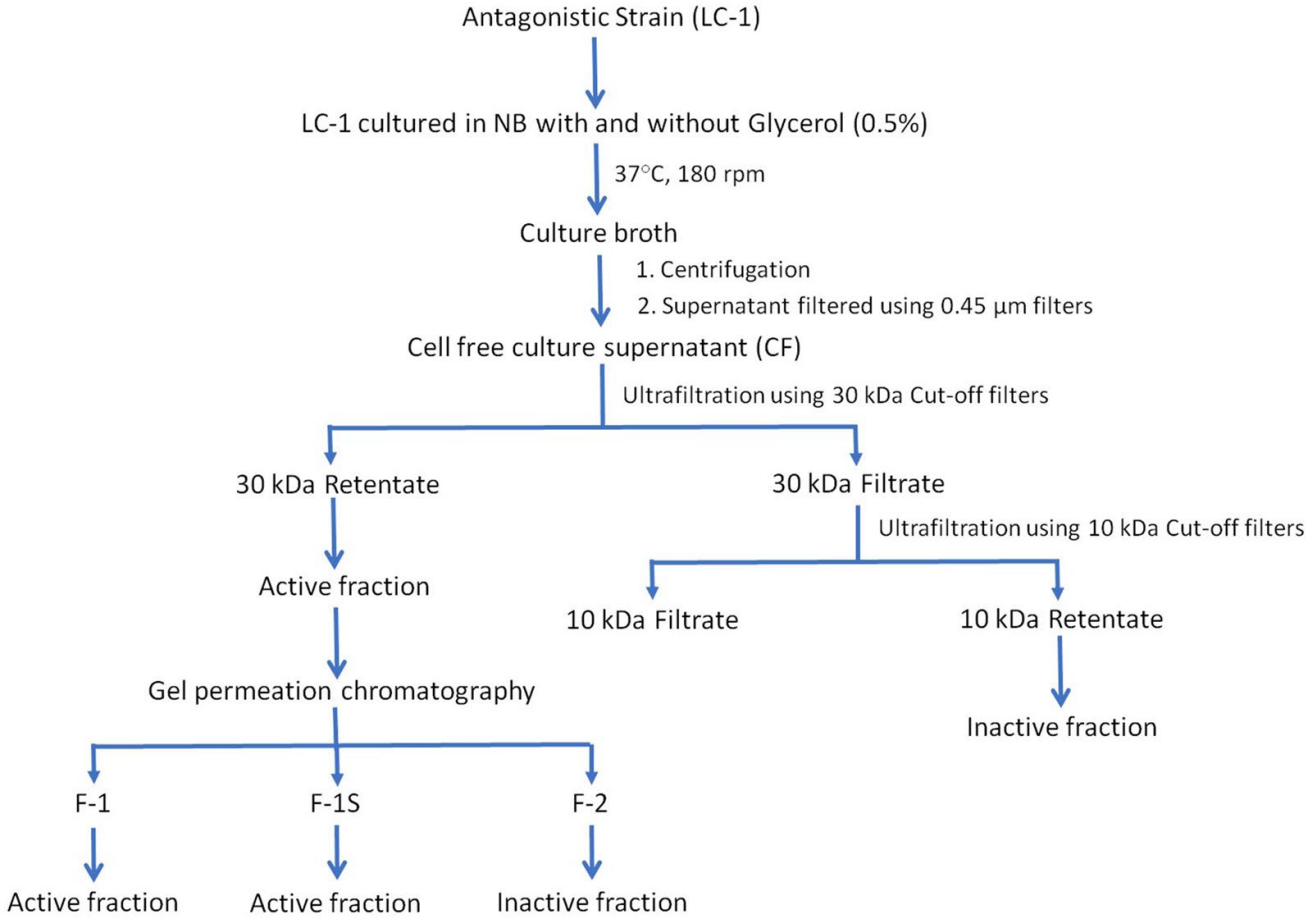
Flow chart outlining production and partial purification of anti-mycobacterial/s from LC-1. LC-1: lab contaminating strain; NB: nutrient broth; F-1: Fraction-1; F-1S: F-1 Shoulder fraction; F-2: Fraction-2

G+ and G− were size fractionated sequentially using Amicon Ultra-15 centrifugal filter device (Millipore, USA) with cut-off of 30 kDa and 10 kDa [56]. Briefly, the 30 kDa retentate (30RG+ and 30RG−) and 30 kDa filtrate (30FG+ and 30FG−) were first produced. The 30 kDa filtrates were then passed through 10 kDa centrifugal filter to produce 10 kDa retentate (10RG+ and 10RG−) and 10 kDa filtrate (10FG+ and 10FG−). All the fractions were filter sterilized using sterile 0.22 µm membrane filters and the protein content was quantified using Bradford [57]. The anti-mycobacterial activity of different fractions (75 µg) was evaluated against *M. bovis* BCG strain by agar-well diffusion assay as described above. The active anti-mycobacterial fraction was subjected to gel permeation chromatography using AKTA Purification system (GE Healthcare Life Sciences). The HiPrep 26/60 Sephacryl S-100 high resolution column was pre-equilibrated and developed, A_280_ peak fractions Fraction-1 (F-1), F-1 shoulder fraction (F-1S) and Fraction-2 (F-2) were collected and concentrated using 3 kDa Amicon centrifugal filter. The concentrated peak fractions were filter sterilized and protein content was quantified as described above. Each fraction (50 µg protein) was individually evaluated for anti-mycobacterial activity against *M. bovis* BCG strain. A flowchart outlining the production and partial purification of anti-mycobacterial fractions of LC-1 is summarized in Fig. 7. The peak fraction exhibiting maximum anti-mycobacterial activity (active fraction) was taken forward.

### Effect of enzyme treatment, temperature and pH on the stability of active fraction

The effect of enzyme treatment, temperature and pH on the stability of the active fraction was evaluated by comparing its residual anti-mycobacterial activity with respect to its untreated control. An aliquot of the active fraction containing 42.5 µg of proteins was treated with enzyme namely Proteinase K and α-chymotrypsin (1 mg/ml final concentration) and controls without enzyme were applied. All the samples were incubated for 4 h at 37 °C and evaluated for activity against *M. bovis* BCG using disc diffusion assay. To determine thermal stability, the active fraction was subjected to 100 °C for 30 and 60 min. The samples were gradually cooled to room temperature and evaluated for anti-mycobacterial activity against *M. bovis* BCG. To evaluate the sensitivity of active fraction to varying pH, it was treated with various buffers (50 mM) having pH ranging from 3.0 to 10.5 which included Sodium citrate buffer (pH: 3.0, 4.5 and 6.0), phosphate buffer (pH: 7.5) and Tris–HCl buffer (pH: 9.0 and 10.5). Untreated controls were prepared by adding sterile deionized water in place of buffers, whereas buffers alone were used as negative control. All the preparations were incubated at 37 °C for 2 h and evaluated for residual bactericidal activity against *M. bovis* BCG using disc diffusion assay. Residual activity was assessed by comparing zone of growth inhibition (in mm) of treated and untreated controls.

### Antimicrobial spectrum of active fraction

In addition to *M. bovis* BCG, the antimicrobial spectrum of partially purified active fraction was also evaluated against *M. smegmatis*, *E. coli*, and *S. aureus* by well/disc diffusion assay as described previously [55]. The assay was performed by applying active fraction containing 50 µg of protein in each well/disc on agar plates seeded with a uniform lawn of these bacterial cultures (as used for testing the antagonistic spectrum of LC-1). The plates were incubated at 37 °C; overnight for *E. coli* and *S. aureus*, 2 days for *M. smegmatis* and 15–21 days for *M. bovis* BCG. Antimicrobial potential of active fraction was assessed by observing zones of inhibition around the disc/well. The effect of active fraction F-1 on the growth of *M. bovis* BCG in liquid culture was also assessed. Briefly, an exponential culture of *M. bovis* BCG (O.D_595nm_ 0.0125) grown in 7H9-ADS broth was exposed to different concentrations of F-1 (75, 150 and 300 µg/ml of protein) in a clear bottom black 96-well plate at 37 °C and O.D_595nm_ was recorded at 72 h post treatment. Phosphate buffer (20 mM, pH 7.2) was used as buffer control. ΔO.D_595nm_ for test and control cultures was plotted and analysed.

### MALDI-TOF MS/MS analysis

Before MALDI-TOF MS/MS analysis, the active cut-off fraction 30RG− fractionated by gel permeation chromatography and its different elution fractions, F-1, F-1S and F-2 were subjected to Laemmli SDS-PAGE (15% w/v) analysis and Coomassie Brilliant Blue R-250 staining.

MALDI-TOF mass spectrometry was carried out to identify protein/s present in anti-mycobacterial fractions, F-1 and F-1S. The samples were processed using standardized protocol at Advanced Technology Platform Centre (ATPC), Regional Centre of Biotechnology, Faridabad, Haryana [58].

- Processing of F-1: Briefly, 1 × 1 mm gel was cut to excise the protein band at the size ~ 35 kDa and destained in wash solution (50% acetonitrile, 50 mM ammonium bicarbonate, pH 8.0) to completely remove Coomassie Brilliant Blue stain. It was followed by dehydration in 100% acetonitrile (ACN) and then subjected to reduction (5 mM DTT, 100 mM ammonium bicarbonate pH 8.0) and alkylation (10 mM iodoacetamide, 100 mM ammonium bicarbonate). The Alkylating solution was pipetted out and cleaned using wash solution followed by dehydration as above. The gel was finally rehydrated in 50 mM ammonium bicarbonate and digested overnight with trypsin (1:50 w/w) at 37 °C [59]. The sample was further extracted using 60% ACN, 0.1% Formic acid and desalted in Zip-Tip C18 column using 0.1% Formic acid (FA) in 5% ACN and finally eluted in 0.1% FA and 70% ACN. The eluted sample was dried and reconstituted in 10 µl of 2% ACN and 0.1% FA and further mixed with a matrix solution, α-cyano-4-hydroxycinnamic acid (5 mg/ml in 80% ACN and 0.1% TFA). One microlitre was spotted onto sample plate in triplicate and allowed to dry.
- Processing of F-1S: 35 μg protein of F-2 fraction was subjected to acetone precipitation. The pellet was resuspended in 100 mM ammonium bicarbonate buffer (pH 8.0) followed by reduction (5 mM DTT) and alkylation (10 mM iodoacetamide). The sample was further digested with trypsin (1:50 w/w) for overnight at 37 °C. The tryptic digested peptide mixture was further fractionated by nano-LC (eksigent nanoLC 425 SIGMA Aldrich) using Chromolith Caprod RP-18e HR capillary column (150 × 0.1 mm; Merck Millipore) prior to MALDI-TOF MS/MS analysis.

### MALDI-TOF MS/MS analysis of peptides

The processed peptides were subjected to MALDI-TOF MS/MS analysis using 5800 MALDI–TOF/TOF analyser (AB SCIEX) and 4000 Series Explorer software, version 4.0 (AB SCIEX). The Instrument was operated in positive ion mode. The laser power was set between 3100 and 3500 for MS and between 3800 and 4300 for MS/MS acquisition. MALDI peptide spectra were calibrated using matrix ion peak as per international standard. The data obtained under mass spectrometer were searched against bacterial database of protein sequence from NCBInr using the programme MASCOT (http://matrixscience.com) with a parameter of carboxymethylation, deamination and oxidation of cysteine residues allowing up to two missed trypsin cleavage and monoisotopic mass tolerance of 0.5 Da.

## Supplementary Information

**Additional file 1: Figure S1.** Effect of active fraction F-1 on the growth of *M. bovis* BCG in liquid media. **Figure S2.** Amino acid sequences of Staphylococcal proteins in fraction F-1 and F-1S listed in Tables 3 and 4.

## Data Availability

All data generated or analysed during this study are included in this published article.

## Abbreviations

LC-1: Laboratory contaminant
Mtb: *Mycobacterium tuberculosis*
WHO: World Health Organisation
BSL-3: Biosafety level 3
CF: Culture filtrate
MC: Media control
G+ & G−: Culture filtrate obtained by culturing LC-1 in nutrient broth supplemented with Glycerol and without Glycerol respectively
30RG+ & 30RG−: 30 kDa retentate of G+ and G− respectively obtained by ultrafiltration using 30 kDa cut-off filter
30FG+ & 30FG−: 30 kDa filtrate of G+ and G− respectively obtained by ultrafiltration using 30 kDa cut-off filter
10RG+ & 10RG−: 10 kDa retentate of 30FG+ and 30FG− respectively obtained by ultrafiltration using 10 kDa cut-off filter
MALDI-TOF: Matrix-Assisted Laser Desorption/Ionization-Time of Flight
