# Supplementary material for: Anti-mycobacterial activity of heat and pH stable high molecular weight protein(s) secreted by a bacterial laboratory contaminant
Source: Microb Cell Fact. 2022 Jan 29;21:15. doi: 10.1186/s12934-022-01743-2 (PMC8799974; doi:10.1186/s12934-022-01743-2)
Supplement: Supplementary file 1 — Additional file 1: Figure S1. Effect of active fraction F-1 on the growth of M. bovis BCG in liquid media. Figure S2. Amino acid sequences of Staphylococcal proteins in fraction F-1 and F-1S listed in Tables 3 and 4. [file 12934_2022_1743_MOESM1_ESM.pdf]

Additional file 1

Anti-mycobacterial activity of heat and pH stable high molecular weight protein(s) secreted by a bacterial laboratory contaminant.

Md. Sajid Hussain[1,2], Atul Vashist[3$*], Mahadevan Kumar[3β], Neetu Kumra Taneja[3#], Uma Shankar Gautam[3λ], Seema Dwivedi[1], Jaya Sivaswami Tyagi[3] and Rajesh Kumar Gupta[2*]

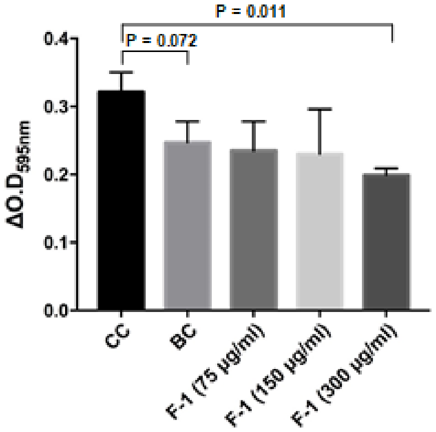

Fig. S1: Effect of active fraction F-1 on the growth of *M. bovis* BCG in liquid media. *M. bovis* BCG culture in duplicates ($O.D_{595nm}$ 0.0125) was treated in presence or absence of either Phosphate buffer or different concentrations of active fraction F-1 at 37°C for 72 hours in 7H9-ADS liquid media. $\Delta O.D_{595nm}$ values (Final O.D – Initial O.D) of F-1 treated and Phosphate buffer treated culture were plotted and compared to that of untreated culture. BC and CC respectively stand for Phosphate buffer control and untreated culture control.

Fig. S2: Amino acid sequences of Staphylococcal proteins in fraction F-1 and F-1S as listed in Tables 3 and 4 (Peptide sequences identified by MALDI-TOF MS/MS analysis are indicated in bold Red.)

1. **SphX**, Mol. Wt.: 34.934 kDa, pI value: 5.98, Peptide coverage: 41%

MKKWQLVGTTVLGATVLLGACGGGNSGGSGSGDGKDLEGSAKGEGSSTVAPIVEKLNEKWAK**DHPSA**
TISSGQAGTGAGFEKFIAGETDFSQASRPIKDEEKQKLEDKNIKYKEFK**IAQDGVTVAVNKDNDFVK**ELS
KDQLKKIYSGEAKTWKDVDSSWPNKEIK**AFSPNSSHGTYDFWEEEVMDKQDIK**AQKNGDTNVIVQSV
EK**NKESIGYFGYNFYK**QNKDKLKEVKIKGDDGKSVEPTKK**TIQDGSYPLSRPLFLYVKEKSLEDNDVMRE**
FLKFTLDDKGKSAEDAGYVASPSKVYKDEIKELDKYKKDSDK

2. **Thioredoxin reductase**, Mol. Wt.: 35.097 kDa, pI value: 6.81, Peptide coverage: 14%

MKKWQLVGTTVLGASVLLGACGGGDSSGSGSGDGKDLKGEAKGEGSSTVAPIVEKLNEKWAKDHPNA
TISSGQAGTGAGFEK**FIAGETDFSQASRPIKDEEK**QKLEDKDIKYKEFK**IAQDGVTVAVNKDNDFVK**ELS
KDQLKKIYNGEAKTWKDVNSSWPDKKIKAFSPNSSHGTYDFFEEEVMDKEDIKAEKNGDTNVIVQSVEK
NKEGIGYFGYNFYEQNKDKLKEVKIKDDKGKTTEPTKKTIKDGSYALSRPLFLYVKEK**SLKDNDVMR**EFIKF
TLEDKGKSAEDA GYVASPEKTYKDELKDLKKY DKKSDK

3. **Alkylphosphonate ABC transporter**, Mol. Wt.: 35.038 kDa, pI value: 9.13, Peptide coverage: 3%

MKNFKYLFVLMLAVIVFAAACGNSSSLDNQKNASNDSDSKSGGYKPKELTVQFVPSQNAGTLEAKAKPL
EKLLSKELGIPVKVSVSTNYNTIVEAMKSKKVDVGFLPPTAYTLAHDQK**AADLLLQAQR**FGVKEDGSASK
ELVDSYKSEILVKKDSKIKSLKDLKGKKIALQDVTSTAGYTFPLAMLKNEAGINATKDMKIVNVKGHDQAV
ISLLNGDVDAAAVFNDARNTVKKDQPNVFKDTRILKLTQAIPNDTISVRPDMDKDFQEKLKKAFIDIAKSK
EGHKIISEVYSHEGYTETKDSNFDIVREYEKLVKDMK

4. **Hypothetical protein SH0318**, Mol. Wt.: 35.104 kDa, pI value: 8.83, Peptide coverage: 3%

MKQMKYLLVLALTVIIFAAACGNNSSLDSNNKSSDSGSDSGKDGYKPKELTVQFVPSQNADTLEAKAKPL
EKLLSKKLDIPVKVSVSTNYNTIVEAMKSKKVDVGFLPPTAYTLAHDQK**AADLLLKAQR**YGVNEDGSNSK
KLVDDYKSEILVKKDSGINSLKDLKGKKIALQDVTSTAGYTFPISTLKEEAGIDATKDMKIVNVKGHDQAVI
SLLNGDVDAAAVFQDARTIVKKDQPNVFKDTKIIKLTEPIPNDTISVRPDMDKAFQDKLKKAFKDIAKTKE
GHKIISE VYSHEGYTDAKDSDFDIVRK YEKQVQDMK

5. **Lipase**, Mol. Wt., 62.029 kDa, pI value, 5.19, Peptide coverage: 19%

MTNESPTEEKTTTQTTTAETNTSSTKDNTSKTTDSHSTKTSQSTPLTESKELTPSKTQQDSNVNQDAIKN
NDVEAPSTGKTNDTVDTTETKQTVTAKQPTDPAAPTVNTLNTKDEAISIDGTTNETVGHKDAKDQKNSQ
AGLETLANNAVATTNNTSQQQGTTETKDQTNKVAKQGQYKNQDPIILVHGFNGFTDDINPNVLSHYW
GGDKLNIRQDLEQNGYNAYEASISAFGSNYDR**AVELYYYIK**GGTVDYGAAHAERYGHERYGKTYEGVYK
DWQPG QKVHLVGHSM GGQTVR**QLEELLRNGSQEEI EYQK**THGGDI SPLLQGGHDN MVSSITTLGT
PHNGTHAADELGNEALVRQVVFDLGKRLGNKNSR**VDFGLSQWGLKQQPGESYISYLLR**VKNSK**LWQS**
KDNGFYDLTRDGATDLNRKTSLNPNIVYKMYTAEATHPTLIGKQK**ADYNMFLPFTVTGNVIGK**ATEKW
RENDGLVSVISSQHPFNQAYTEATDTNQK**GIWQVTPTKHDWDHVDFVGQDSTDTKR**SREELQQFWY
DLADDLVQTEALTSTNE

6. **Dihydrolipoyl dehydrogenase**, Mol. Wt.: 49.716 kDa, pI value: 4.84, Peptide coverage: 19%

M**VVGDFPIETDTIVIGAGPGGYVAAIR**AAQLGQKVTIVEKGDLGGVCLNVGCIPSKALLHASHRFVEAQ
HSENLGIIAESVSLKFDKVQEFKQSVVNKLTGGVEGLLKGNKVDIVRGEAYFVDENSLRVMDEKSAQTYN
FK**NAIIATGSRPIEIPNFEFGKR**VIDSTGALNLQEVPGKLVVVGGGYIGSELGTVFANFGSEVTILEGAKEIL
GGFEKQMVQPVKKGMKEKGVEIITEAMAKNAEETENGVKVTYEAKGEEK**TIEADYVLVTVGRRPNTDE**
LGLEELGLKFADRGLLEVDKQSRTSIK**NIYAIGDIVPGLPLAHK**ASYEGKVAAEVISGQASEVDYIGMPAVC
FTEPELAQVGYTEAQAKEEGLDFKASKFPYAANGRALSLDDTTGFVKLITLKEDDTVIGAQVAGNGASDII
SELGLAI EAGMNAEDIALTVHAHPTLGEMTMEVAEKAIGLPIHTM

7. **Phosphopyruvate hydratase**, Mol. Wt.: 47.154 kDa, pI value: 4.54, Peptide coverage: 29%

M**PIITDVYAR**EVLDSR**GNPTVEVEVLTESGAFGR**ALVPSGASTGEHEAVELRDGDKSRYSGKGVTKAVE
NVNEIIAPEIVEGEFSVLDQVSIDKMMIQLDGTPNKGKLGANAILGVSIAVARAAADLLGQPLYKYFGGFN
GKQLPVPMMNIVNGGSHSDAPIAFQEFMILPTGAESFKEALR**WGAEIFHNLK**SILSER**GLETAVGDEGG**
FAPRFDGTEDAVETIIKAIEKAGYKPGEDVFLGFDCASSEFYENGVYDYTKFEGEHGAKRSAAEQVDYLEE
LISKYPIITIEDGMDENDWDGWKQLTDR**IGDKVQLVGDDLFVTNTEILSRGIEQGIGNSILIKVNQIGTLT**
ETFDAIEMAQKAGYTAVVSHRSGETEDTTIADIAVATNAGQIKTGSLSRTDRIAK**YNQLLRIEDELFETAK**
FDGIKSFYNLDK

8. **Alkaline Phosphatase 3**, Mol. Wt.: 53.395 kDa, pI value: 5.37, Peptide coverage: 18%

MSFINKFGKTTVATSILAASVLGTTHVSFASGAGQGAQDQQGQNGDYMAIGNTKNPK**NVIFMVGDG**
MGPSYNSAYRYYADNPNTKELDQTAFDKYLKGTNRTNPNDPKENVTDSAAGGTAFATGHK**TYNGSIS**
VDNDKKPLKSVLEYAKEQGKSTGLVTTAEVTDATPAVYAAHVDDRDKKDDIAQQFYNDKINGQHKVDV
ILGGGSKYFGKENGHLTEKFQKDGYDYVTNKTDLANSKSDQVLGLFAEKNMPLQIDAPQQNPLLADME
ESALSKLEKNDKGFFLMVEGASIDKSGHPNDITGVMSEMSGFDKAFQNAIDYAK**NHKDTLVVATADHS**
TGGLSIAKGKEYVWNPDAIHKMKHSGSYMTEQIAKGKDPETVINEGYGVDFPKKQLDKVKNAAKELKD
VKDKAKNDDDPKIAEATTQLQDAIQKPINDESHTGWTTYGHTGEDVNTYAYGPGADKFRGNIDNTDSA
KNIFDFFGQDVTSNQNQQ

9. **Dihydrolipoamide dehydrogenase**, Mol. Wt.: 49.421 kDa, pI value: 4.95, Peptide coverage: 12%

M**VVGDFPIETDTIVIGAGPGGYVAAIR**AAQLGQKVTIVEKGNLGGVCLNVGCIPSKALLHASHRFVEAQ
HSENLGVIAESVSLNFQKVQEFKSSVVNKLTGGVEGLLKGNKVNIVKGEAYFVDNNSLRVMDEKSAQTY
NFK**NAIIATGSRPIEIPNFKFGKR**VIDSTGALNLQEVPGKLVVVGGGYIGSELGTAFANFGSEVTILEGAKD
ILGGFEKQMTQPVKKGMKEKGVEIVTEAMAKSAEETDNGVKVTYEAKGEEK**TIEADYVLVTVGR**RPNT
DELGLEELGVKFADRGLLEVDKQSRTSISNIYAIGDIVPGLPLAHKASYEAKVAAEAIDGQAAEVDYIGMP
AVCFTEPELATVGYSEAQAKEEGLAIKASKFPYAANGRALSLDDTNGFVKLITLKEDDTLIGAQVVGTGAS
DIISELGLAIEAGMNAEDIALTIHAHPTLGEMTMEAAEKAIGYPIHTM

10. **Purine nucleoside Phosphorylase**, Mol. Wt.: 25.905 kDa, pI value: 4.79, Peptide coverage: 17%

MTNGTPHIQPNGAKIAKTVLMPGDPLR**AKYIADNFLENVEQFNEVR**NMFGYTGTYKGKEISVMGSGM
GIPSIGIYSYELYNFFDVDTIIRIGSCGALQENVNLYDVIIAQAASTNSNYVDQFNIPGHFAPIADFELITKAK
QVADDIGAVTHVGNILSSDTFYNADKHFNDSWKNMGILGIEMESAGLYLNAIHAGKK**ALGIFTVSDHILR**
DEATSAEERQTSFTQMMEIALEIAE

11. **DNA binding protein HU 1,** Mol. Wt.: 9.650 kDa, pI value: 9.52, Peptide coverage: 58%

MNKTDLINAVAEQADLTKKEAGSAVDAVFESIQNSLSKGEKVQLIGFGNFEVRERAARKGRNPQTGKE
IEIPASKVPAFKAGKALKDAVK

12. **Alkaline Phosphatase III precursor,** Mol. Wt.: 53.385 kDa, pI value: 5.22, Peptide coverage: 5%

MSFINKFGKTTVASSILAASVLGTTHVSFASGSGEGNQGQQGQNEDYMAIGNTKNPKNVIFMVGDGM
GPAYNSAYRYYADNPNTKELDQTAFDKYLKGTNRTNPNDPKENVTDSAAGGTAFATGYKTYNGAISVD
NNKKPLKSVLEKAKELGK**STGIVTTAEVTDATPAVYAAHVDDR**DKKDEIAQQFYNDKINGQHKADVILG
GGSKYFGKENGNLTDKFQKDGYDYVTNKDELANSQSDQVLGLFSEKNMPLQIDAPQSNPLLVDMENSA
LSKLEKNDKGFFLMVEGASIDKSGHPNDITGVMSEMGGFEKAFQNAIDYSNKHKDTLVVATADHSTGG
LSIAKGKDYVWNPEAIHKMKHSGSYMTKQIADGKDPEKVINDGYGFEFPSKQMDKVKDATKELKEAQD
KAKSEDDEKVAAATTKLQDAIQKPINDESHTGWTTYGHTGEDVNTYAHGPSENKFAGNNDNTDSAKNI
FDFFNNDVTSNQNQQ

13. **2-Phospho-D-glycerate hydrolase (enolase)**, Mol. Wt.: 47.240 kDa, pI value: 4.63, Peptide coverage: 10%

M**PIITDVYAR**EVLDSR**GNPTVEVEVLTESGAFGR**ALVPSGASTGEYEAVELRDGDKDRYLGKGVTKAVE
NVNEKIAPEIVEGEFSVLDQVSIDKMMIQLDGTENKGKLGANAILGVSIAVARAAADLLGQPLYKYLGGF
NGKQLPVPMMNIVNGGSHSDAPIAFQEFMILPVGADSFKESLRWGAEIFHNLAKILKKRGLETAVGDEG
GFAPKFHGTEDAVDTILEAIKAVGLEPGKDVYLGFDIASSEFYENGVYDYTKFEGPEGAKRTSAEQVDYLE
ELVNKYPIITIEDGMDENDWDGWKQLTDRLGKKVQLVGDDLFVTNTKKLSEGIEKGIGNSILIK**VNQIGTL**
TETFDAIQMAQKAGYTAVISHRSGETEDTTIADIAVATNAGQIKTGSLSRTDRIAKYNQLLRIEDELYETAE
FDGIHSFYNLDK
